# Systematic statistical analysis of change in SEIzure interVAL with diazepam nasal spray supports this novel clinical endpoint for immediate‐use seizure medication

**DOI:** 10.1002/epi.70222

**Published:** 2026-04-01

**Authors:** Wesley T. Kerr, Katherine N. McFarlane, Leock Y. Ngo, Enrique Carrazana, Adrian L. Rabinowicz

**Affiliations:** ^1^ Division of Epilepsy, Department of Neurology University of Pittsburgh Pittsburgh Pennsylvania USA; ^2^ Department of Biomedical Informatics University of Pittsburgh Pittsburgh Pennsylvania USA; ^3^ Neurelis San Diego California USA; ^4^ John A. Burns School of Medicine University of Hawaii Honolulu Hawaii USA; ^5^ Center for Molecular Biology and Biotechnology, Charles E. Schmidt College of Science Florida Atlantic University Boca Raton Florida USA

**Keywords:** benzodiazepine, epilepsy, intranasal, rescue, seizure cluster

## Abstract

**Objective:**

Appropriate endpoints for daily antiseizure medications may differ from those for intermittent, immediate‐use seizure medications (ISMs). The observed interval between seizure clusters over time (SEIzure interVAL [SEIVAL]) has been proposed as a novel effectiveness endpoint for ISMs. SEIVAL was initially identified pragmatically. This post hoc analysis evaluated how the study timeline and participant characteristics could impact the measurement of SEIVAL using data from an open‐label study of intermittent treatment with diazepam nasal spray.

**Methods:**

This analysis includes data from a long‐term, phase 3 safety study of diazepam nasal spray, which enrolled patients aged 6–65 years with epilepsy and seizure clusters (NCT02721069). Study timeline and participant characteristic choices were evaluated systematically to determine the impact on effect size, and thereby statistical power, using data regarding individual treated seizure clusters. In this strategy, the date of the first treated seizure cluster constituted day 1 of the baseline period; consecutive 70‐day periods were used for analysis. The change in SEIVAL for each patient was calculated using each measured SEIVAL for that patient in the primary outcome period (days 71–140) minus that patient's mean SEIVAL in the baseline period (days 1–70) with mixed effects linear regression.

**Results:**

Mean SEIVAL increased from 13 days at baseline (days 1–70) to 24 days in the primary outcome period (days 71–140, *p* < .0001). SEIVAL lengthening was seen in adults, adolescents (ages 6–17 years), and children (ages 6–12 years), with further lengthening past 140 days in adolescents and children. SEIVAL lengthened more in participants with <1 SEIVAL per 2 weeks at baseline than higher SEIVAL frequency.

**Significance:**

This systematically planned analysis expands on and reinforces the previous pragmatic analysis that introduced the SEIVAL metric. The present analysis provides a novel framework for future studies of intermittently administered ISMs to treat seizure clusters such as diazepam nasal spray.

**Key points**

- Appropriate metrics for the effectiveness of intermittent therapies may be different from those for daily antiseizure medications.
- SEIzure interVAL (SEIVAL), the number of days between treated seizure clusters, has potential as an effectiveness endpoint.
- This systematic SEIVAL analysis demonstrates an increase in SEIVAL over time, consistent with an analysis employing a pragmatic approach.

## 1 INTRODUCTION

Epilepsy is defined, in part, as an enduring predisposition for unprovoked and often unpredictable seizures.^1^ Epilepsy treatment focuses on achieving seizure freedom, but ≥30% of patients with epilepsy have drug‐resistant epilepsy.^2^, ^3^ Although each individual seizure has profound impact, intermittent episodes of seizure(s) called seizure clusters may have even greater impact.^4^, ^5^ Untreated seizure clusters can transition into status epilepticus,^6^ which can result in additional neuronal injury. Additionally, seizure clusters are a driver of emergency department visits and hospitalizations. “Rescue” medication for seizure clusters has been associated with improvements in quality of life as well as patient and caregiver satisfaction.^7^, ^8^, ^9^

Diazepam nasal spray is approved by the US Food and Drug Administration for acute treatment of intermittent, stereotypic episodes of frequent seizure activity (i.e., seizure clusters, acute repetitive seizures) that are distinct from a patient's usual seizure pattern in patients with epilepsy aged ≥2 years.^10^ In a long‐term, phase 3 safety study in patients with epilepsy aged 6 to 65 years (*N* = 163), diazepam nasal spray was well tolerated and showed effectiveness, with only a single dose used to control approximately 87% of seizure clusters for a 24‐h period.^11^

Although the effectiveness and safety of immediate‐use seizure medications (ISMs) for the acute treatment of seizures is well established, there may be secondary long‐term benefits to improving acute seizure treatment.^12^, ^13^, ^14^, ^15^, ^16^ One proposed long‐term effectiveness outcome that maintains the focus on seizure clusters is the interval in days between seizure clusters, called SEIVAL (SEIzure interVAL).^12^ Intervals may be a better outcome measure than frequency for seizure clusters, because they are less frequent (e.g., 2‐month interval between clusters) as compared to individual seizures (e.g., four seizures per month).^17^, ^18^, ^19^ Lengthening SEIVAL may be a natural long‐term effectiveness outcome measure of both daily antiseizure medications (ASMs) and ISMs. Because the immediate pharmacologic effect of ISMs^20^ is much shorter than the outcome of SEIVAL, a significant lengthening of SEIVAL with ISM treatment may indicate an enduring modification of the underlying disease that leads to seizure clusters.^8^, ^21^

There is preliminary evidence that ISMs may lengthen SEIVAL. In a placebo‐controlled preclinical study in rats, the preclinical analog of ISMs was associated with an increase in time between seizures in a cluster and reduced seizure severity, findings that endured in the 2‐week period after ISM treatments.^21^

Post hoc analysis of treated seizure‐cluster data from patient diaries from the phase 3 safety study of diazepam nasal spray in patients with epilepsy aged 6–65 years demonstrated that the mean SEIVAL increased substantially over the course of 1 year, from 14.8 days during the first 90‐day period of the study (days 1–90) to 35.8 days during the fourth 90‐day period (days 271–360).^8^ That analysis and the 90‐day period duration were chosen pragmatically.^8^ Although a blinded placebo‐controlled study is generally desirable for establishing efficacy, randomization of participants to placebo for 1 year in a placebo‐controlled study would not be ethical. However, data available from the open‐label safety study can provide insights into benefits and limitations to inform future study designs. Key analysis parameters include the timeline of evaluation from baseline through the outcome period, the minimum and maximum SEIVAL required for eligibility, whether the outcome was consistent across a sufficiently large participant population, and the statistical approach that maximizes statistical power.

The objective of this post hoc analysis of an open‐label safety study was to more comprehensively evaluate the impact of each study design and participant characteristic choice on the longitudinal lengthening of SEIVAL after intermittent treatment of seizure clusters with diazepam nasal spray. In addition, because this endpoint should be considered for future treatment studies, this work provides a potential path forward for those studies.

## 2 MATERIALS AND METHODS

### 2.1 Study design

This study is a reanalysis of the multicenter phase 3 safety study of diazepam nasal spray that was conducted from April 2016 to July 2020 (NCT02721069).^11^ The evaluation of safety, tolerability, and other aspects of the design and analysis of the original study was described previously.^11^ Written informed consent was provided by participants or parents/guardians before study participation.

Participants aged 6–65 years had a diagnosis of focal or generalized epilepsy with motor seizures or seizures with a clear alteration of awareness; occurrence of seizures despite a stable ASM regimen that, in the opinion of the investigator, might have needed intermittent benzodiazepine intervention (i.e., ISM) for seizure control at least, on average, once every other month; and participation of a care partner who could administer study medication.^11^ History of status epilepticus was permitted. There was no restriction on concomitant use of benzodiazepines (e.g., clobazam), including use as maintenance therapy.^22^ Other inclusion and exclusion criteria were summarized in the original study.^11^ The study included a 12‐month treatment period after which participants could elect to continue therapy at the discretion of the investigator.^11^

Participants and care partners were instructed to administer 5‐, 10‐, 15‐, or 20‐mg doses of diazepam nasal spray based on age and weight for treatment of frequent seizure activity distinct from the participant's usual seizure pattern.^11^ If needed during the study, caregivers were instructed to administer a second dose 4–12 h after the first dose. Dosing could be adjusted by the investigator for effectiveness or safety reasons. The proportion of seizure clusters for which second doses were administered within 24 h of the initial dose was used as a proxy for effectiveness. Time of onset of each treated seizure cluster, the time diazepam nasal spray was administered, and the time the seizure cluster stopped were recorded in a patient diary.^11^

In these post hoc analyses, SEIVAL was defined as the time in days between two treated seizure clusters, calculated from the end of the first treated seizure cluster to the beginning of the second treated seizure cluster. Only seizure clusters treated with diazepam nasal spray were included in this analysis. Although SEIVAL was measured in days, fractional days were calculated based on the hour and minute of these events. We evaluated whether re‐treatments reflected short SEIVALs or whether these additional treatments for a single seizure cluster should be excluded. Due to variable definitions of cluster duration across participants, re‐treatment was defined by less than 24 h between treatments. For seizure clusters with re‐treatment, SEIVAL was calculated as the time interval from the end of the last treated seizure in the re‐treated seizure cluster to the beginning of the second seizure cluster. To enable the analysis of change in SEIVAL, we required participants to have ≥2 SEIVALs during the study period.

### 2.2 Study timeline

The effectiveness outcome of SEIVAL is based on testing the hypothesis that SEIVAL lengthened when seizure clusters were treated, compared with when they were not treated. SEIVAL is a long‐term outcome and is not focused on the immediate effect of treatment. Therefore, compared with ASM clinical trials that evaluate pretreatment baseline, a study of SEIVAL could be shortened by starting measurement after the first treated seizure cluster. In the prior SEIVAL analysis, the baseline period was pragmatically chosen to be the first 90 days after the first treated seizure cluster.^8^ The last pretreatment seizure cluster was not recorded.

**FIGURE 1:**
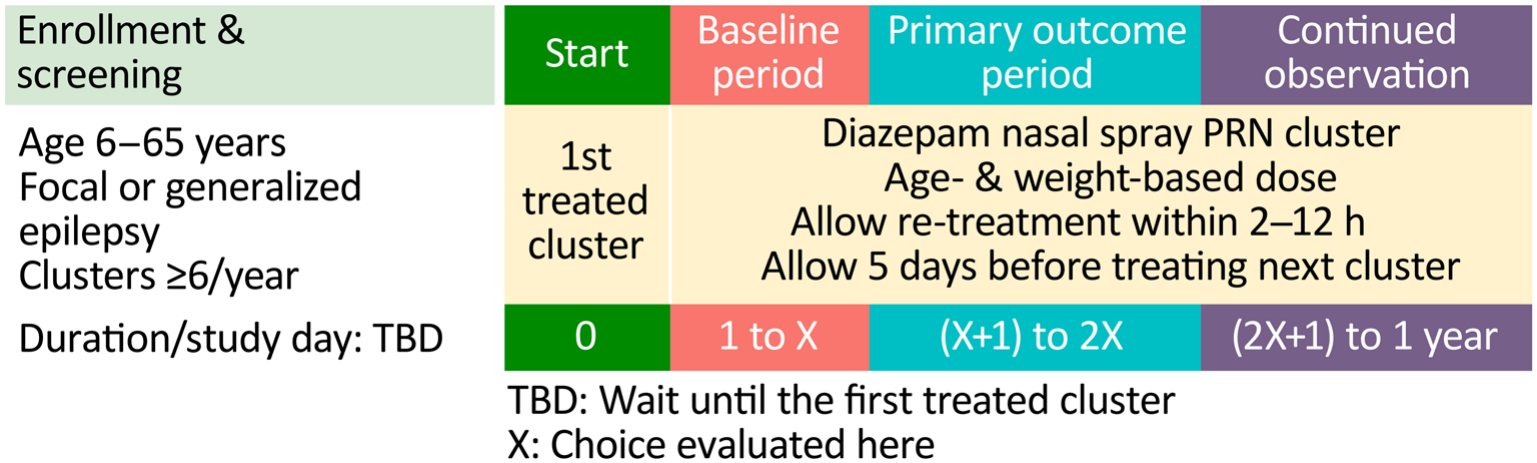
Study timeline to evaluate interval between seizure clusters (SEIVAL). PRN, pro re nata (as needed).

The primary outcome period was defined as the earliest period after the baseline period where the effect of SEIVAL could be reliably measured (Figure 1). The primary outcome period was not required to start immediately after the baseline period. The prior analysis evaluated sequential 90‐day periods to allow for inclusion of a sufficient number of participants who had ≥1 SEIVAL in each period.^8^ To address the potential impact of retention bias, a subanalysis was performed on a consistent cohort of participants with ≥1 SEIVAL in each of the first four 90‐day periods up to 360 days after the first treated seizure cluster.^8^

### 2.3 Analysis of multiple parameters on SEIVAL

To assist with shaping the design of future studies that evaluate the hypothesis that SEIVAL lengthens with treatment, we evaluated multiple analytical parameters. We compared each option and favored the choice that maximized the effect size, which was defined as the magnitude of SEIVAL in the primary outcome period compared with the baseline period divided by the SE of the difference in SEIVAL between those periods. There is a direct relationship between effect size and statistical power. That analytical option using paired measurements of SEIVAL where each participant was compared with their individual baseline generally has improved statistical power compared with other approaches.

#### 2.3.1 Selection of SEIVAL timing

For a SEIVAL spanning from the baseline into the primary outcome period, we evaluated whether the period of the SEIVAL should be determined based on the time of the first or second treated seizure cluster. As described above, we also evaluated whether re‐treatments within 24 h reflected short SEIVALs or should be excluded by measuring SEIVAL from the end of the re‐treated seizure cluster, even if the seizure cluster stopped between the first and re‐treated seizure(s).

#### 2.3.2 Measurement of SEIVAL in each study period

In the prior analysis,^8^ the SEIVAL during a study period was calculated from the average of all SEIVALs that occurred. The interval between two events commonly is considered either exponentially or Poisson distributed; therefore, we also evaluated a “robust” average SEIVAL, which was the average of the log of SEIVAL.^23^ A change in the robust average SEIVAL would reflect a proportional change in SEIVAL (e.g., SEIVAL was 50% shorter). Because participants could have multiple SEIVALs during each period, we evaluated whether incorporating those repeated measurements of SEIVAL could improve statistical power using multilevel mixed effects regression modeling that accounted for the inherent intraparticipant correlation of repeated measurements of SEIVAL.^24^ Owing to low recruitment from some sites, we did not use additional multilevel terms to address site‐level variability. We considered all differences with *p*‐values < .05 to be statistically significant.

#### 2.3.3 Selection of baseline and primary outcome period length

The best study timeline would measure the lengthening of SEIVAL both quickly and reliably (Figure 1). With the goal of shortening the trial compared with the prior SEIVAL analysis that used 90‐day periods,^8^ we evaluated all choices of baseline and primary outcome period length, indicated as X in Figure 1, from 1 day through 90 days. If the baseline period was too short, then there may be too few measurements of SEIVAL to reliably compare with the primary outcome period. If the baseline period was too long, then SEIVAL may lengthen within the baseline, which could blur the distinction between baseline and primary outcome periods. The primary outcome period also needed to be long enough that a sufficient number of participants had ≥1 SEIVAL within the period and, ideally, sufficient SEIVALs to measure the difference between baseline and primary outcome period. If treatment had a large beneficial impact on SEIVAL, then participants' SEIVAL may be too long to experience a SEIVAL during the primary outcome period. However, if the combination of baseline and primary outcome were too long, then sample size may be limited by attrition (e.g., as in the prior consistent cohort analysis^8^). In sensitivity analyses, we evaluated whether there was benefit of an interval period between baseline and primary outcome periods and whether baseline and primary outcome periods should be different lengths.

An alternative to prespecifying the duration of the primary outcome period is to use a time‐to‐event design.^25^ We evaluated the time‐to‐event design where each participant's baseline period was used to define an average number of seizure clusters per 28‐day month.^26^, ^27^ The length of the subsequent primary outcome period would then be defined as the time to baseline monthly seizure cluster count (T‐BMSC) or the maximum duration of the study period, whichever occurred first.^25^ The impact of treatment on T‐BMSC was measured with Cox proportional hazards, with the baseline period serving as a simulated placebo. As BMSC is defined by the 28‐day baseline period, if SEIVAL in the outcome period matched the baseline period, then T‐BMSC in the outcome period would also be only 28 days.

#### 2.3.4 Association of SEIVAL lengthening with baseline SEIVAL

In clinical trials of ASMs, key eligibility criteria are the minimum and maximum seizure frequency.^28^ To be eligible for this open‐label study, participants were required to be expected to need ISM ≥6 times per year.^11^ To evaluate the association of SEIVAL lengthening with baseline SEIVAL, we grouped participants based on the number of SEIVALs recorded during the baseline period.

#### 2.3.5 Patterns of SEIVAL lengthening in children compared to adults

Doses of diazepam nasal spray were adjusted for patient age (as well as weight). We also evaluated whether the timeline of SEIVAL lengthening differed between pediatric participants aged ≥6 years and adults aged ≥18 years.

## 3 RESULTS

In the original phase 3 study, 163 participants received ≥1 dose of diazepam nasal spray, and 120 participants had ≥2 SEIVALs (Figure S1).^8^, ^11^ Participant characteristics mirror the original study and are reproduced in Table S1.^11^

### 3.1 Selection of baseline and primary outcome period length

**FIGURE 2:**
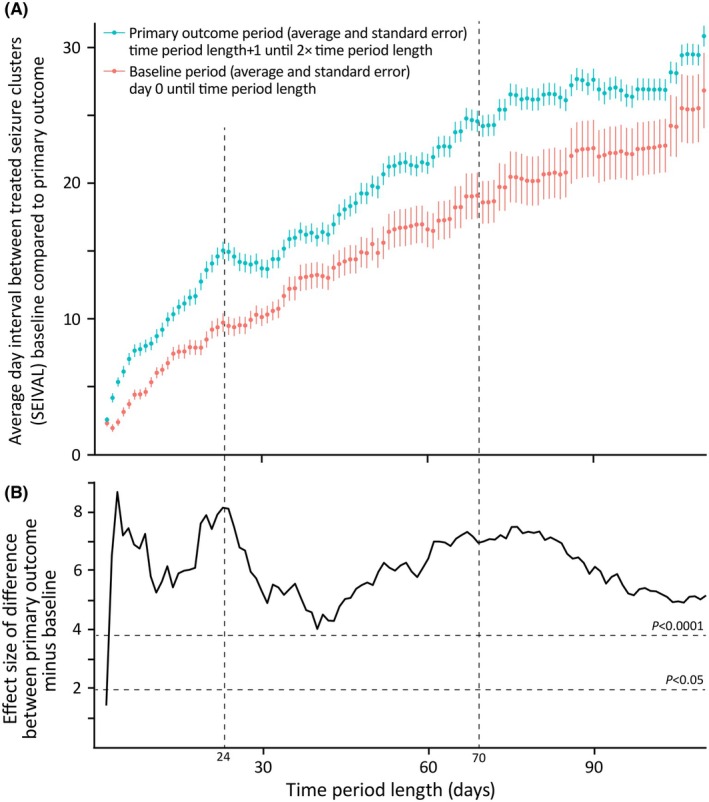
Association of period length with interval between treated seizure clusters (SEIVAL) lengthening. (A) Average SEIVAL increased when time periods were longer. Error bars reflect SE. (B) Effect size of the difference between the two sequential periods of baseline (day 0 until time period length [X in Figure 1]) and primary outcome (day time period length + 1 until day 2× time period length) had peaks at 24 and 70 days (vertical dotted lines). SEIVALs were defined based on when the second treated seizure clustered occurred. Effect size is defined as the difference of the average divided by the SE of the difference. Vertical dotted lines reflect effect sizes corresponding to a significant effect (*p* < .05 and *p* < .0001) using these data and these study timelines.

The relationship between the average SEIVAL during the baseline and primary outcome periods, based on the length of the periods, is illustrated in Figure 2A. As expected, the average SEIVAL increased with longer periods. Effect size (Figure 2B) was maximized early for sequential 24‐day periods, but small variations in the length of the period were associated with lower effect size. There was a broad local maximum of effect size around 70‐day periods where average SEIVAL during baseline was 13 days compared to 24 days during the primary outcome period (*p* < .0001). All 120 participants had ≥1 SEIVAL during the 10‐week baseline period. Subsequently, 88% (106/120) of participants had ≥1 SEIVAL during the following 70‐day primary outcome period.

**FIGURE 3:**
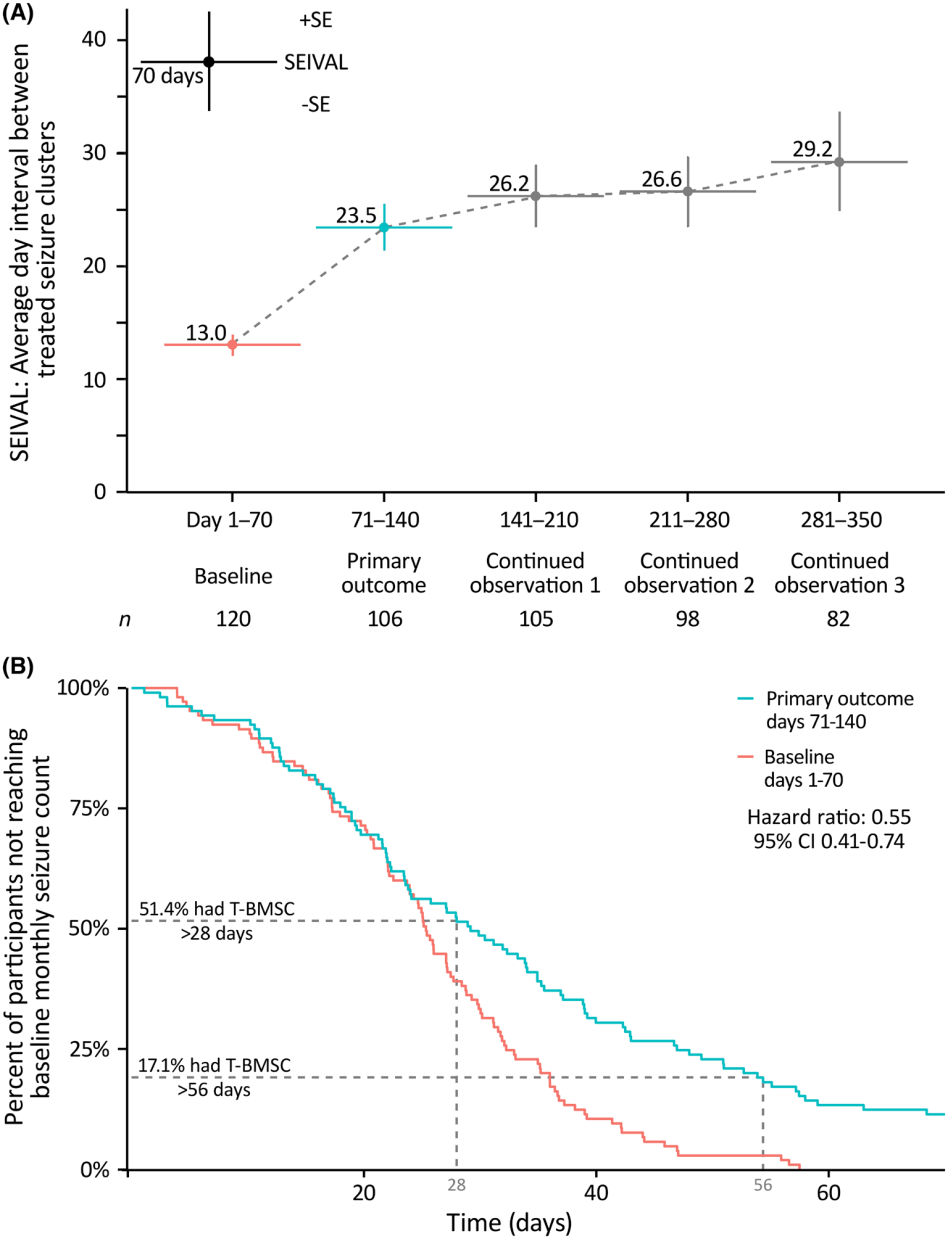
Timeline of change in average interval between seizure clusters (SEIVAL). (A) The average SEIVAL was shorter in the baseline period (days 1–70) compared with all subsequent periods. There was less change in SEIVAL after the primary outcome period (days 71–140). Error bars reflect SE. (B) Time to baseline monthly seizure cluster count (T‐BMSC) during the primary outcome period (days 71–140) compared with the simulated placebo of the baseline period. Time to SEIVAL lengthening may be delayed past 10 weeks in some participants. n, number of participants with a SEIVAL in the period. CI, confidence interval.

For comparison, the previous consistent cohort of participants enrolled for ≥1 year with ≥1 SEIVAL during each 90‐day period retained 73% (87/120) of participants.^8^ Figure 3A illustrates the magnitude of extending the evaluation of SEIVAL into additional 10‐week periods over 1 year. Allowing an interval between the baseline and primary outcome periods or lengthening the primary outcome period past 10 weeks did not result in improved effect size (results not shown). Although considering re‐treatments within 24 h as SEIVALs lowered the average SEIVAL, there was no substantial shift in effect size (effect size of 7.04 with re‐treatments and 7.05 without [*p* < .0001 for both]). When SEIVALs were assigned to periods based on the time of the first treated seizure cluster, there was no significant change in SEIVAL (*p* = .32).

When a time‐to‐event design was tested, there was a significant lengthening of SEIVAL in the primary outcome period compared with the simulated placebo of the baseline period (hazard ratio = .55, SE = .15, 95% confidence interval = .41–.74, *p* < .0001). The effect size of this analysis was 10.5, which was higher than the 70‐day analysis, despite the lack of divergence between the primary outcome period and the simulated placebo of the baseline period before 21 days in the Kaplan–Meier curve (Figure 3B).

### 3.2 Patterns of SEIVAL lengthening in children compared to adults

**FIGURE 4:**
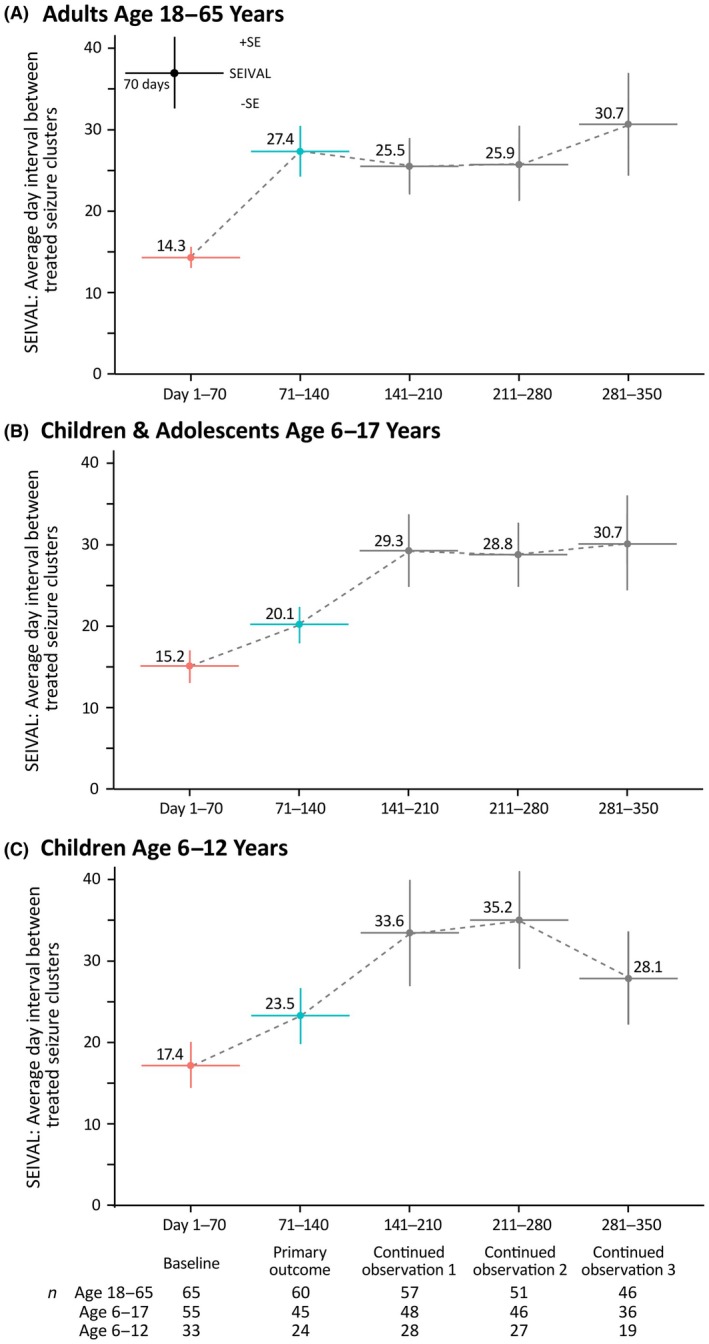
Average interval between seizure clusters (SEIVAL) patterns in adult (A) and pediatric participants (B, C). SEIVAL lengthened over the course of the study for all participants. Bars reflect average SEIVAL over 70‐day periods, and error bars reflect SE. Compared with adults, participants younger than 18 years had less lengthening of SEIVAL in the primary outcome period, followed by a greater lengthening of SEIVAL in the first continued observation period (days 141–210; see also Table S2). n, number of participants with a SEIVAL in the interval.

Figure 4 illustrates the patterns in adults (18–65 years), children and adolescents (6–17 years), and children (6–11 years) based on 70‐day periods. SEIVAL was longer in the primary outcome period than baseline and the primary outcome period in all three cohorts and statistically significant in the adult cohort (age = 18–65 years, *n* = 60, *p* = .016; Table S2). Compared with adults, SEIVAL was initially smaller and not statistically significant for children (age = 6–17 years, *n* = 45, *p* = .34; age = 6–11 years, *n* = 24, *p* = .42), followed by a greater, statistically significant lengthening in the first continued observation period (days 141–210: age = 6–17 years, *n* = 48, *p* = .0065; age = 6–11 years, *n* = 28, *p* = .025), which remained significant in subsequent periods.

### 3.3 Association of SEIVAL lengthening with baseline SEIVAL

**FIGURE 5:**
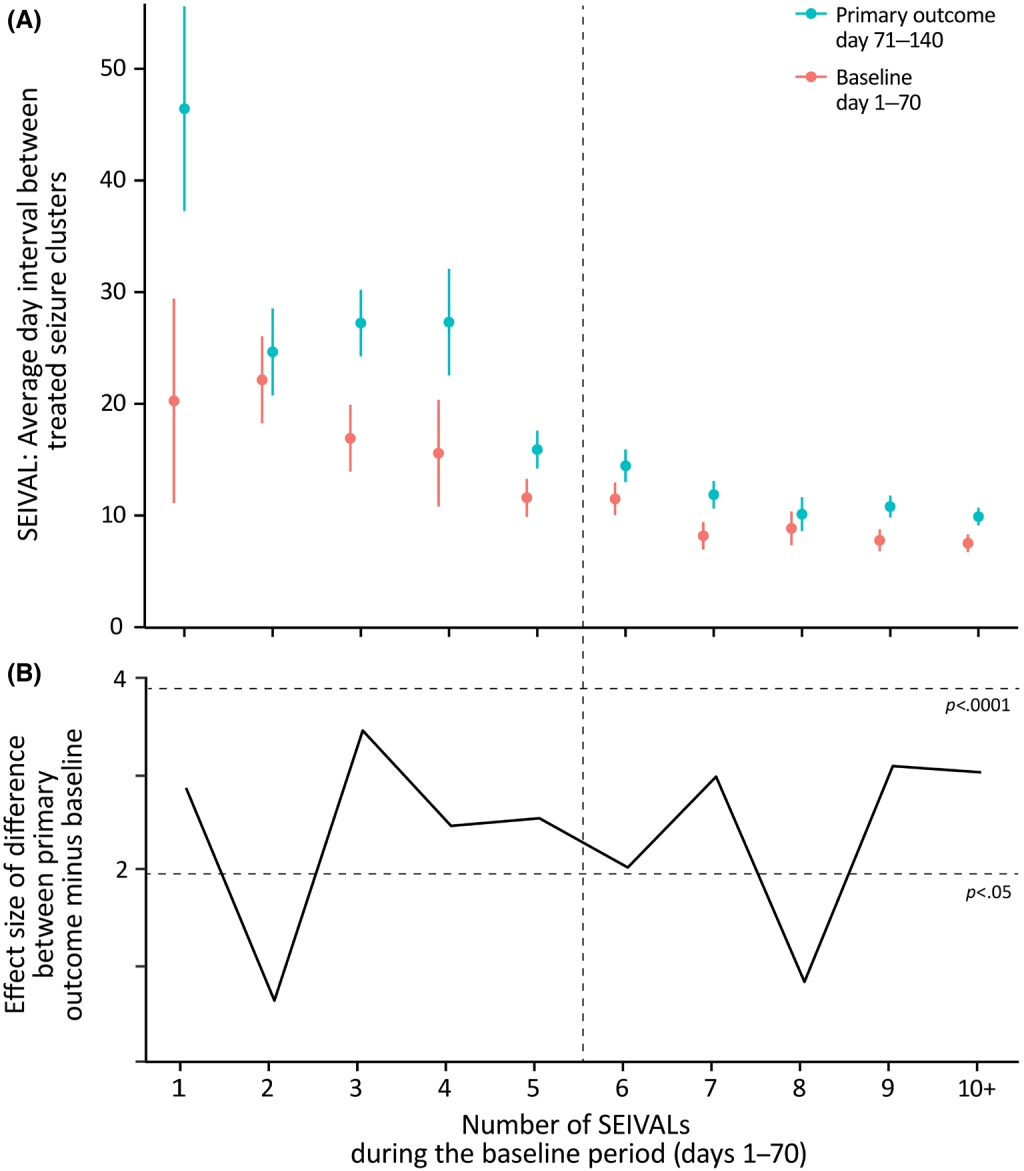
Interval between seizure clusters (SEIVAL) lengthened more in participants with fewer baseline SEIVALs. Association of mean baseline SEIVAL count and change in SEIVAL from baseline to primary outcome periods. Error bars reflect SE.

Participants with 1–5 SEIVALs per 70 days (average ≤1 treatment per 14 days) during the baseline period had a larger change in SEIVAL (17 days) during the primary outcome period than patients with >5 SEIVALs (6.5 days; Figure 5A). The effect size of SEIVAL lengthening was generally consistent across baseline SEIVAL counts (Figure 5B), although SEIVAL counts of 2 and 8 were not statistically different from baseline.

### 3.4 Measurement of SEIVAL in each study period

The pragmatic approach of evaluating average SEIVAL using 90‐day periods had greater or similar effect size compared to other statistical models. Mixed effects models incorporating repeated measurements of SEIVAL did not substantially alter results (baseline average SEIVAL = 19 days, primary outcome SEIVAL = 24 days, *p* < .0001). The effect size of the log‐transformed “robust” average SEIVAL was less than the untransformed average (baseline robust average = 10 days vs. primary outcome robust average = 13 days, *p* < .0001).

## 4 DISCUSSION

This post hoc analysis used data from a phase 3 open‐label safety study of the ISM diazepam nasal spray to provide insights into how the interval between seizure clusters, SEIVAL, may be used to measure an enduring effect of treatments on seizure clusters^21^ in subsequent studies. Compared with the previous pragmatic analysis of 90‐day periods over 1 year,^8^ these results showed that a significant lengthening of SEIVAL could be observed with a posttreatment baseline period of 10 weeks (70 days) followed immediately by a primary outcome period of 10 weeks. Although there was a delayed time to SEIVAL lengthening in some participants (e.g., children and adolescents, time‐to‐event analysis), 10‐week periods would maximize the effect size. SEIVAL lengthened in all participants, irrespective of baseline SEIVAL count, but the magnitude of SEIVAL lengthening was greater in participants with ≤1 treated seizure cluster every 2 weeks. This analysis provides meaningful practical and statistical insights to guide future studies that use SEIVAL as an effectiveness endpoint, and lengthening of SEIVAL continuing over time can be seen as a meaningful change, with statistically significant lengthening potentially indicating enduring modification of disease. Also, the earlier 90‐day SEIVAL analysis included a subgroup of adult patients (*n* = 35) in the consistent cohort who completed the Quality of Life in Epilepsy‐31‐P instrument and had clinically meaningful improvement on the Social Functioning subscale (7.7 days [minimally important change = 4.0 days]),^8^ suggesting that the statistical change was accompanied by a clinically meaningful improvement for patients. Additionally, these results further characterize the beneficial association of intermittent nasal diazepam with lengthening SEIVAL.

This SEIVAL analysis used data from the same phase 3 safety study that enrolled patients with seizure clusters intermittently treated with diazepam nasal spray as the previous pragmatic SEIVAL analysis. The earlier analysis looked at mean SEIVAL in 90‐day periods over time and found a consistent and statistically significant pattern of lengthening SEIVAL among the overall group with SEIVALs, the consistent cohort, and subgroups by age.^8^ The current systematic statistical analysis of change in SEIVAL examining patient‐level SEIVAL data over 70‐day periods found a similar pattern of lengthening SEIVAL over time. In both analyses, an approximate doubling of mean SEIVAL was seen across time (13.9–26.8 days in the consistent cohort with re‐treatments eliminated in the earlier analysis^8^). Also, both pediatric and adult patients experienced SEIVAL lengthening over time in both analyses (patients 6–17 years and ≥18 years old in the consistent cohort of the earlier analysis^8^).

A substantial lengthening in average SEIVAL from 13 to 24 days could be detected reliably in a 20‐week study with two 10‐week periods (baseline and primary outcome periods). This shorter duration was a substantial improvement upon the prior analysis of SEIVAL, which focused on four 90‐day periods over the course of 1 year.^8^ Although the effect of intermittent nasal diazepam was observed using even shorter durations, 10 weeks appeared to reflect a broad local maximum that balanced the importance of short periods with longer periods to measure SEIVAL more reliably. The local maximum of effect size demonstrated that some participants had substantially longer SEIVALs after 10 weeks of treatment.

In children and adolescents, lengthening was observed during the primary outcome period, but even further lengthening was observed in the next 10‐week period. Additionally, the time‐to‐event analyses demonstrated that some participants' SEIVAL may not change until ≥3 weeks.^25^ Therefore, the choice of 10‐week periods may reflect a compromise between measuring the lengthening of SEIVAL both in participants with early and those with later onset of the effect.

In addition, we observed even greater lengthening of SEIVAL in participants who treated seizure clusters less than once every 2 weeks in the baseline period. The pharmacologic effect of ISMs is limited to hours^20^; therefore, people who require an ISM more than once every 2 weeks may warrant change in their long‐term daily antiseizure treatments. The effect size of SEIVAL lengthening was also similar in participants with higher and lower baseline SEIVAL counts.

One statistical explanation for a greater magnitude of effect in participants with lower baseline count was that SEIVAL may have lengthened proportionally (e.g., 1.8‐fold) as compared with linearly (e.g., increase by 10.5 days). However, a log‐transform statistical approach showed no meaningful improvement in effect size. Additionally, multilevel modeling approaches^24^ that incorporated multiple measurements of SEIVAL by the same participant within the same period did not substantially improve the effect size or statistical power.

Although re‐treatments within 24 h of a seizure cluster could be considered very short SEIVALs, the inclusion of re‐treatments did not have a substantial impact on the study's ability to observe the average lengthening of SEIVAL, which was similar to the previous analysis.^8^ Only 13% (485/3853) of seizure clusters were re‐treated.^11^ The lack of impact of re‐treatments on the measurement of SEIVAL may indicate that a similar proportion of re‐treatments occurred during the baseline and primary outcome phases. Nevertheless, we propose that analyses of SEIVAL exclude re‐treatments within 24 h, because they are likely to be clinically different from the interval between seizure clusters, SEIVAL, which was typically >2 weeks.

Although these post hoc analyses aimed to inform subsequent studies, they are limited by the use of an internal baseline period. Paired testing has superior statistical power to two independent samples, but the internal comparison did not account for longitudinal changes unrelated to treatment. Placebo‐controlled studies or in silico approaches are needed to evaluate whether the lengthening in SEIVAL may be attributed to regression to the mean,^29^ reduced enthusiasm for treatment (however, mean duration on study was 17.4 months and retention was 71.8%^11^; untreated seizure clusters were not recorded), or other unmeasured confounding factors.^30^ Additionally, future prospective studies could evaluate associations with other patient characteristics that may identify why SEIVAL lengthened more for participants with lower cluster frequency. Previous analyses of the impact of diazepam nasal spray for seizure clusters demonstrated that change in concomitant medications, prior intermittent benzodiazepine use or daily benzodiazepine treatment (e.g., clobazam), and developmental epileptic encephalopathies (identified post hoc) did not reduce its effectiveness in cluster cessation.^8^, ^22^, ^31^ Future studies also may evaluate whether those and other participant characteristics may be associated with SEIVAL lengthening.

## 5 CONCLUSIONS

In this post hoc analysis of a long‐term safety study, patients who used intermittent diazepam nasal spray for acute treatment of seizure clusters experienced an increase in SEIVAL from the first 10 weeks of treatment to the second 10 weeks of treatment. Even though the direct pharmacokinetic impact of intermittent diazepam diminishes within hours of treatment, the increase in SEIVAL was sustained or improved upon with continued treatment for the remainder of the year. These analyses further characterize how study timeline and participant characteristic choices may impact the effect size, and thereby statistical power, of potential future studies that may use SEIVAL as an effectiveness outcome.

## AUTHOR CONTRIBUTIONS

All authors provided substantial contributions to conception and design, drafting, and revising the manuscript critically for important intellectual content, and all authors gave final approval of the version to be submitted.

## FUNDING INFORMATION

This study was funded by Neurelis, Inc. (San Diego, California). W.T.K.'s research time was funded by the National Institute of Neurological Disorders and Stroke (K23NS135134).

## CONFLICT OF INTEREST STATEMENT

W.T.K. has received compensation as Associate Editor of *Epilepsia*; writes review articles for *Medlink Neurology*; is a paid consultant for SK Life Sciences, UCB Pharmaceuticals, Jazz Pharmaceuticals, Azurity, Acuta, Ventus, Capsida, Epygenix, Biohaven Pharmaceuticals, the Epilepsy Study Consortium, Cerebral Therapeutics, Neurelis, Inc., Noema, EpiTel, QurAlis, Neurona, NeuroPace, and Rapport; and has collaborative or data use agreements with Eisai, Janssen, Johnson & Johnson, Praxis, Radius Health, and GSK. K.N.M. is a paid consultant for Neurelis, Inc. and NeuroPace. L.Y.N. is an employee of and has received stock options from Neurelis, Inc. E.C. is an employee of and has received stock and stock options from Neurelis, Inc. A.L.R. is an employee of and has received stock options from Neurelis, Inc. We confirm that we have read the Journal's position on issues involved in ethical publication and affirm that this report is consistent with those guidelines.

## ETHICS APPROVAL STATEMENT

The original study protocol, informed consent form, and other relevant study documentation were approved by ethics committees or institutional review boards at each site before study initiation.

## Supporting information


TABLE S1

## Data Availability

All relevant data are within the paper.
