# Supplementary material for: Systematic statistical analysis of change in SEIzure interVAL with diazepam nasal spray supports this novel clinical endpoint for immediate‐use seizure medication
Source: Epilepsia. 2026 Apr 1;67(7):3432–43. doi: 10.1002/epi.70222 (PMC13361021; doi:10.1002/epi.70222)
Supplement: Supplementary file 1 — TABLE S1 [file EPI-67-3432-s001.docx]

**Supplemental Table 1: Participant Characteristics^11^**

| **Variable** | **N=163** |
| --- | --- |
| Baseline demographics | |
| Age, mean (SD), y | 23.1 (15.1) |
| Range, y | 6-65 |
| 6–11 y, n (%) | 45 (27.6) |
| ≥12 y, n (%) | 118 (72.4) |
| Sex, n (%) |  |
| Male | 74 (45.4) |
| Female | 89 (54.6) |
| Race, n (%) |  |
| White | 134 (82.2) |
| Black/African American | 16 (9.8) |
| Asian | 4 (2.5) |
| Native Hawaiian/Pacific Islander | 5 (3.1) |
| Other | 4 (2.5) |
| Height, mean (SD), cm | 151.6 (24.8)^a^ |
| Weight, mean (SD), kg | 60.2 (33.6)^b^ |

^a^n=159.

^b^n=162.

**Supplemental Table 2: SEIVAL within each period and age group**

The patterns in the average interval between seizure clusters (SEIVAL) in each study period are visualized in **Figures 3 and 4**. The SE for the baseline period reflects the SE of average SEIVAL. The SE for each period reflects the SE of the change in SEIVAL in each period compared with the baseline period. All time periods were compared with the baseline period; therefore, effect size or *P* value were not applicable for the baseline period.

| **Period** | **SEIVAL** | **SE** | **Effect size** | ***P*** |
| --- | --- | --- | --- | --- |
| All participants |  | | | |
| Baseline (days 1-70) | 13.0 | 2.6 |  |  |
| Primary outcome (days 71-140) | 23.5 | 3.8 | 2.8 | 0.0059 |
| Continued observation period 1 (days 141-210) | 26.2 | 3.8 | 3.5 | 0.00053 |
| Continued observation period 2 (days 211-280) | 26.6 | 3.9 | 3.5 | 0.00047 |
| Continued observation period 3 (days 281-350) | 29.2 | 4.1 | 4.0 | 7.2 x10^-5^ |
| Adults (18 to 65 y) |  | | | |
| Baseline (days 1-70) | 14.3 | 3.7 |  |  |
| Primary outcome (days 71-140) | 27.4 | 5.4 | 2.4 | 0.016 |
| Continued observation period 1 (days 141-210) | 25.5 | 5.5 | 2.0 | 0.042 |
| Continued observation period 2 (days 211-280) | 25.9 | 5.6 | 2.1 | 0.041 |
| Continued observation period 3 (days 281-350) | 30.7 | 5.8 | 2.8 | 0.0052 |
| Children & Adolescents (6-17 y) |  | | | |
| Baseline (days 1-70) | 15.2 | 3.5 |  |  |
| Primary outcome (days 71-140) | 20.1 | 5.2 | 0.9 | 0.34 |
| Continued observation period 1 (days 141-210) | 29.3 | 5.1 | 2.7 | 0.0065 |
| Continued observation period 2 (days 211-280) | 28.8 | 5.2 | 2.6 | 0.0095 |
| Continued observation period 3 (days 281-350) | 30.1 | 5.6 | 2.7 | 0.0078 |
| Children (6-11 y) |  | | | |
| Baseline (days 1-70) | 17.4 | 4.9 |  |  |
| Primary outcome (days 71-140) | 23.5 | 7.5 | 0.8 | 0.42 |
| Continued observation period 1 (days 141-210) | 33.6 | 7.2 | 2.3 | 0.025 |
| Continued observation period 2 (days 211-280) | 35.2 | 7.2 | 2.5 | 0.015 |
| Continued observation period 3 (days 281-350) | 28.1 | 8.0 | 1.3 | 0.19 |

**Supplemental Figure 1. STROBE Flow Chart^8^**


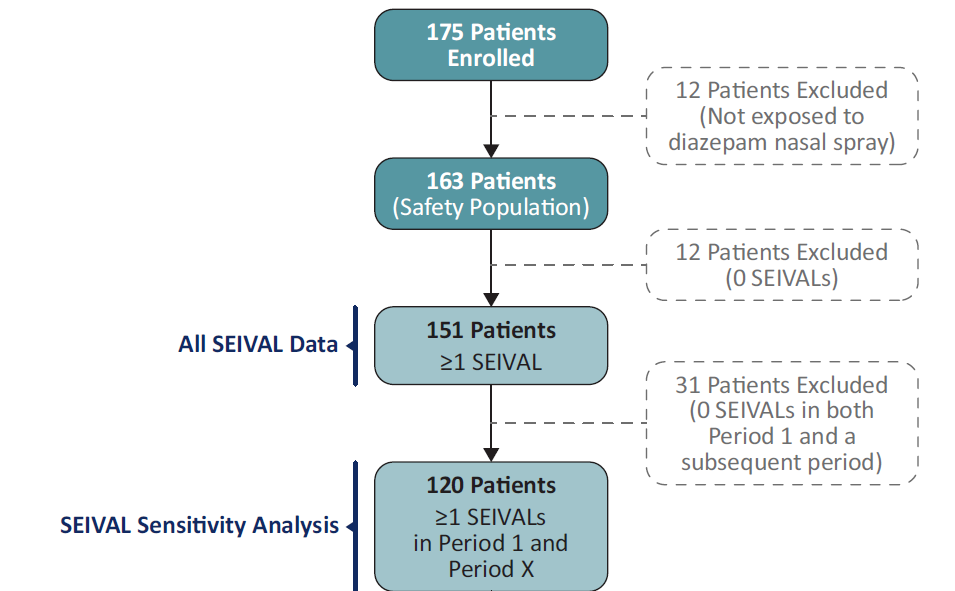
 Abbreviations: SEIVAL, interval between seizure clusters; STROBE, Strengthening the Reporting of Observational Studies in Epidemiology.
